# Seawater Acidification and Elevated Temperature Affect Gene Expression Patterns of the Pearl Oyster *Pinctada fucata*

**DOI:** 10.1371/journal.pone.0033679

**Published:** 2012-03-16

**Authors:** Wenguang Liu, Xiande Huang, Jianshi Lin, Maoxian He

**Affiliations:** Key Laboratory of Marine Bio-resources Sustainable Utilization, South China Sea Institute of Oceanology, Chinese Academy of Sciences, Guangzhou, China; University of Vigo, Spain

## Abstract

Oceanic uptake of anthropogenic carbon dioxide results in decrease in seawater pH and increase in temperature. In this study, we demonstrated the synergistic effects of elevated seawater temperature and declined seawater pH on gene expression patterns of *aspein*, *calmodulin*, *nacrein*, *she-7-F10* and *hsp70* in the pearl oyster *Pinctada fucata*. Under ‘business-as-usual’ scenarios, four treatments were examined: (1) ambient pH (8.10) and ambient temperature (27°C) (control condition), (2) ambient pH and elevated temperature (+3°C), (3) declined pH (7.70) and ambient temperature, (4) declined pH and elevated temperature. The results showed that under warming and acidic seawater conditions, expression of *aspein* and *calmodulin* showed no significant differences among different time point in condition 8.10 T. But the levels of *aspein* and *calmodulin* in conditions 8.10 T+3, 7.70 T and 7.70 T+3, and levels of *nacrein*, *she-7-F10* in all the four treatments changed significantly. Low pH and pH×temperature interaction influenced the expression of *aspein* and *calmodulin* significantly after hours 48 and 96. Significant effects of low pH and pH×temperature interaction on the expression of *nacrein* were observed at hour 96. The expression level of *she-7-F10* was affected significantly by pH after hours 48 and 96. The expression of *hsp70* was significantly affected by temperature, pH, temperature×pH interaction at hour 6, and by temperature×pH interaction at hour 24. This study suggested that declined pH and pH×temperature interaction induced down regulation of calcification related genes, and the interaction between declined seawater pH and elevated temperature caused up regulation of *hsp70* in *P. facata*. These results demonstrate that the declined seawater pH and elevated temperature will impact the physiological process, and potentially the adaptability of *P. fucata* to future warming and acidified ocean.

## Introduction

Increasing concentrations of CO_2_ in the atmosphere are causing the ocean to become warmer and acidify [1]. Global surface temperatures rose by 0.76°C and global seawater pH decreased by 0.1 unit due to increasing CO_2_ emissions since the industrial revolution [2]. Under ‘business as usual’ scenarios, the ocean is predicted to increase in sea surface temperature by 1–4°C and decrease in ocean pH by 0.3–0.4 units by the year 2100 [3], [4]. Ocean absorbing of emitted CO_2_ lead to profound changes in the seawater carbonate chemistry with decrease in calcite, aragonite saturation state and seawater carbonate ions. These changes have been identified as a great threat to marine organisms, particularly to calcifying organisms [5], [6].

Effects of ocean acidification on calcification of marine organisms have been a focus in recent studies [7]. Decreasing in seawater pH has negative effects on calcification rate of organisms like coral *Stylophora pistillata* [8], echinoderm *Amphiura filiformis* [9] and molluscs *Limacina helicina* and *Crassostrea gigas* [10], [11]. However, other studies reported that calcification rate showed no significant change in *Mytilus edulis*, and increased in *Littorina littorea* and *Sepia officinalis* when exposed to low seawater pH [12], [13]. Hence, marine organisms' response to carbonate system variations is diverse.

It is predicted that the oceans will warm and acidify simultaneously. Therefore, studies that include both decreased seawater pH and increased temperature will provide a more realistic assessment of marine organism's responses to future environmental change, than studies limited to a single factor [14]. In studies examining the synergistic impacts of declined seawater pH and elevated temperature on marine organisms, Reynaud et al. [15] found no reduction in calcification in the coral *S. pistallata* when reared at reduced seawater pH but a 50% reduction in calcification when reared at declined seawater pH and elevated temperature. Metzger et al. [16] showed that the crab *Cancer pagurus* was more sensitive to increased temperature under low pH conditions. Byrne et al. [17] demonstrated that exposure of the abalone *Haliotis coccoradiata* and sea urchin *Heliocidaris erythrogramma* to warming (+2°C to 4°C) and acidification (pH 7.6–7.8) resulted in unshelled larvae and abnormal juveniles. Martin et al. [18] reported that the death of algae *Lithophyllum cabiochae* was observed only under elevated temperature and was two- to threefold higher under elevated *p*CO_2_. They also found that net calcification of *L. cabiochae* decreased by 50% when both temperature and *p*CO_2_ were elevated while no effect was found under elevated temperature and lower pH alone.

To date, studies into the consequences of interactive effects of warming and acidification on animals has been limited to early development or calcification. But more research is needed to understand the core physiological mechanisms behind calcification to assess better the sensitivity of these organisms to ocean warming and acidification. Organisms regulate a number of cellular processes during stressful events, and the regulation and modulation of gene expression can be one of the most rapid and sensitive responses to environmental stress [19], [20]. Assessing the expression pattern of genes responsible for calcification under ocean warming and acidify conditions may be a way to assess the potential impacts of climate change on marine organisms.

Pressure to reduce CO_2_ emissions in response to the threat of climate change has led to governments seeking new options for carbon capture and storage (CCS). Several approaches have been suggested to mitigate the future atmospheric CO_2_ concentration. The most important are ocean sequestration of carbon dioxide [21]. Ocean sequestration may directly affect marine life in the selected dumping sites. Immersion in CO_2_-laden, acidic seawater from CO_2_ injection poses physiological challenges to marine animals that respond by tolerance, compensation, or death. Little is known about molecular mechanism of marine organisms to tolerate seawater acidification [22].

The pearl oyster *Pinctada fucata* is distributed along the southern coasts of China and Japan. It is a species of economic importance for pearl production, approximately a quarter of pearl production (about US$160 million) is from the cultured *P. fucata* [23]. It also plays an important role in the food web as well as in the cycling of carbon and calcium carbonate (CaCO_3_) [24]. Pearl formation and shell growth is a highly controlled biomineralization process [25], [26]. Shell matrix proteins such as Aspein, Nacrein, Calmodulin and She-7-F10 are believed to play a key role in the CaCO_3_ crystal polymorphism (calcite and aragonite) and the microstructures of pearl and shell layers [27]. The *aspein*, encoding an unusually acidic protein, might be a key factor for the formation of the calcitic prismatic layer of *P. fucata* [28]. Calmodulin is a ubiquitous intracellular mediator of calcium signaling and some recent studies showed that calmodulin play an important role in the regulation of the uptake and transport of calcium in biomineralization [29]. Nacrein was believed to mediate both HCO_3_^−^ and Ca^2+^ concentrations, and thus is deeply involved in CaCO_3_ crystallization [30]. Nacrein has been used as a marker to evaluate cellular metabolism during biomineralization. Its production increases with increasing calcium concentration and means that a higher biomineralization rate is occurring [31]. She-7-F10 shared high levels of identity with shell matrix structural proteins and was thought to be involved in the process of shell biomineralization [32]. Hsp70 comprise a group of highly conserved proteins that have general protective function in all living organisms [33]. It has been reported that *P. fucata* has the ability to express Hsp70 in response to stressful stimuli [34].

This study investigates the synergistic effects of seawater warming and declined pH on gene expression patterns of *calmodulin*, *nacrein*, *aspein*, *she-7-F10* and *hsp70* in *P. fucata* to provide the first hand molecular evidence to evaluate the mechanisms for marine mollusc to response to elevated *p*CO_2_ seawater and temperature.

## Results

Total alkalinity (TA) and salinity showed no clear change between treatments throughout the experiment. However, pH, *p*CO_2_, saturation states for aragonite and calcite differed clearly between different pH levels (pH 8.10 vs. 7.70) throughout the experiment (Table 1). The natural temperature in Daya Bay ranged from 26.5 to 27.5 during the experiment. There showed no tank effects among different tanks in each treatment (*p*>0.1).

**Table 1:** Parameters of the carbonate system in each treatment.

8.10 T	T	Sal	pH	TA	*p*CO_2_	DIC	Ωara	Ωcal
0 h	27±0.1	33.36±0.31	8.13±0.02	2187.04±36.04	421.51±22.42	1912.22±20.17	3.14±0.20	4.75±0.21
48 h	27±0.2	33.71±0.12	8.14±0.01	2189.36±41.29	422.16±34.81	1914.75±18.37	3.15±0.09	4.77±0.16
96 h	27±0.1	32.88±0.24	8.14±0.02	2183.68±37.92	421.24±29.17	1915.63±30.13	3.15±0.13	4.76±0.19
7.70 T								
0 h	27±0.2	32.87±0.45	7.70±0.01	2184.11±43.17	1424.40±37.31	2119.23±24.25	1.27±0.07	1.92±0.12
48 h	27±0.3	33.12±0.17	7.69±0.01	2185.73±29.88	1425.48±25.72	2116.44±23.56	1.27±0.09	1.92±0.17
96 h	27±0.1	33.26±0.15	7.70±0.03	2187.39±37.27	1462.75±34.19	2120.57±28.93	1.24±0.12	1.88±0.11
8.10 T+3								
0 h	30±0.1	33.28±0.22	8.14±0.02	2182.76±36.55	427.93±31.55	1868.31±19.08	3.43±0.14	5.16±0.11
48 h	30±0.2	33.31±0.40	8.14±0.01	2180.93±23.61	439.89±27.18	1864.52±26.17	3.37±0.13	5.04±0.13
96 h	30±0.1	32.68±0.51	8.13±0.03	2187.26±29.43	428.86±23.79	1870.24±23.72	3.44±0.15	5.15±0.22
7.70 T+3								
0 h	30±0.1	33.30±0.39	7.70±0.01	2189.56±25.16	1459.84±32.63	2106.77±34.15	1.39±0.09	2.09±0.14
48 h	30±0.2	33.21±0.46	7.71±0.01	2184.11±33.47	1419.84±26.48	2107.62±27.82	1.42±0.12	2.12±0.13
96 h	30±0.2	32.46±0.37	7.68±0.02	2187.68±21.59	1422.21±33.69	2107.80±21.37	1.42±0.17	2.13±0.11

T: temperature (°C), Sal: salinity, TA: total alkalinity (µmol/kg), *p*CO_2_: CO_2_ partial pressure (μatm),

DIC: dissolved inorganic carbon (µmol/kg), Ωara: aragonite saturation state, Ωcal: calcite saturation state.

**Figure 1:**
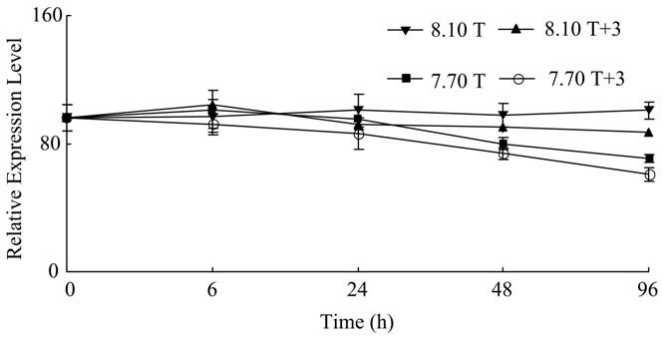
Real-time PCR analysis of expression of *aspein* in response to elevated temperature and declined pH.

The relative expression level of *aspein* fluctuated gradually during hours 0–96 in condition 8.10 T and showed no significant difference among different time point (*p*>0.1). In conditions 8.10 T+3, 7.70 T and 7.70 T+3, the levels decreased significantly to minimum values on hour 96 (*p*<0.1) (Fig. 1, Table 2). The expression of *aspein* was significantly affected by pH and pH×temperature interaction at hours 48 and 96 (*p*<0.1) (Table 3). The expression was significantly lower in condition 7.70 T and 7.70 T+3 than in condition 8.10 T and 8.10 T+3 at hours 48 and 96 (Fig. 1).

**Table 2:** Results of statistical tests performed to test the differences of aspein, calmodulin, nacrein, she-7-F10 and hsp70 in Pinctada fucata among different time point.

Gene	Group	0 h	6 h	24 h	48 h	96 h
*aspein*	8.10 T	96.04±8.14^a^	97.1±10.08^a^	100.65±10.32^a^	97.93±7.34^a^	100.73±5.47^a^
	8.10 T+3	96.04±8.14^a^	104.40±8.52^a^	91.90±7.50^ab^	90.51±3.65^ab^	86.73±2.42^b^
	7.70 T	96.04±8.14^a^	101.30±12.02^a^	94.90±6.06^a^	79.55±4.36^b^	70.48±2.58^c^
	7.70 T+3	96.04±8.14^a^	91.65±6.59^a^	86.40±9.96^b^	73.71±3.09^c^	60.71±3.78^d^
*calmodulin*	8.10 T	0.12±0.01^a^	0.13±0.01^a^	0.12±0.01^a^	0.12±0.02^a^	0.13±0.01^a^
	8.10 T+3	0.12±0.01^a^	0.11±0.01^a^	0.11±0.01^a^	0.11±0.01^a^	0.12±0.01^a^
	7.70 T	0.12±0.01^a^	0.11±0.01^a^	0.09±0.01^b^	0.07±0.01^c^	0.06±0.01^d^
	7.70 T+3	0.12±0.01^a^	0.13±0.01^a^	0.09±0.01^b^	0.06±0.01^c^	0.04±0.01^d^
*nacrein*	8.10 T	6.02±0.50^a^	5.78±0.22^b^	6.26±0.72^c^	5.88±0.49^b^	5.85±0.37^b^
	8.10 T+3	6.02±0.50^a^	6.23±0.28^b^	5.64±0.12^c^	4.88±0.26^d^	4.66±0.30^e^
	7.70 T	6.02±0.50^a^	5.94±0.42^a^	5.20±0.31^b^	4.19±0.38^c^	2.48±0.22^d^
	7.70 T+3	6.02±0.50^a^	5.55±0.52^b^	4.84±0.37^c^	3.89±0.37^d^	1.96±0.11^e^
*she-7-10*	8.10 T	2.61±0.24^a^	2.69±0.20^a^	2.29±0.27^b^	2.31±0.12^b^	2.46±0.07^c^
	8.10 T+3	2.61±0.24^a^	2.53±0.21^a^	2.47±0.10^a^	2.00±0.16^b^	1.98±0.11^b^
	7.70 T	2.61±0.24^a^	2.21±0.15^b^	2.10±0.24^b^	1.67±0.12^c^	0.79±0.05^d^
	7.70 T+3	2.61±0.24^a^	2.02±0.17^b^	1.81±0.18^c^	1.81±0.07^c^	0.21±0.01^d^
*hsp70*	8.10 T	1.30±0.16^a^	2.25±0.14^b^	2.26±0.17^b^	1.79±0.08^c^	1.90±0.11^d^
	8.10 T+3	1.30±0.16^a^	2.99±0.31^b^	2.89±0.26^b^	2.20±0.13^c^	1.42±0.10^a^
	7.70 T	1.30±0.16^a^	3.80±0.33^b^	3.34±0.27^c^	2.37±0.23^d^	1.22±0.09^a^
	7.70 T+3	1.30±0.16^a^	4.30±0.37^b^	4.20±0.18^c^	2.77±0.19^d^	1.03±0.08^a^

Means not sharing the same superscript in each line are significantly different (*p*<0.1).

**Table 3:** Results of statistical tests performed to test the effects of pH and temperature on gene expression patterns of *aspein*, *calmodulin*, *nacrein*, *she-7-F10* and *hsp70* in *Pinctada fucata*.

		6 h			24 h			48 h			96 h		
				Dunn-Sidak test			Dunn-Sidak test			Dunn-Sidak test			Dunn-Sidak test
Source of variation	*df*	F	P	(1) (2) (3) (4)	F	P	(1) (2) (3) (4)	F	P	(1) (2) (3) (4)	F	P	(1) (2) (3) (4)
*aspein*													
pH	1	1.123	0.320		5.524	0.336		7.017	0.029*	a a b b	1.047	0.047*	a a b b
Tem	1	2.072	0.188		2.842	0.181		4.822	0.112		3.151	0.114	
Tem*pH	1	2.130	0.183		7.820	0.111		6.779	0.051*	a a b b	3.208	0.053*	a a b b
*calmodulin*													
pH	1	5.230	0.148		0.953	0.358		3.027	0.013*	a a b b	0.011	0 .001*	a a b b
Tem	1	5.518	0.151		58.026	0.120		10.046	0.120		25.980	0.918	
Tem*pH	1	0.419	0.536		10.460	0.260		3.648	0.093*	a a b b	1.467	0.062*	a a b b
*nacrein*													
pH	1	2.904	0.127		0.274	0.615		2.321	0.166		0.056	0.024*	a a b b
Tem	1	0.577	0.469		46.744	0.973		2.691	0.140		22.591	0.819	
Tem*pH	1	0.978	0.352		2.330	0.209		0.234	0.641		1.864	0.052*	a a b b
*she-7-F10*													
pH	1	0.022	0.885		0.063	0.808		11.986	0.079*	a a b b	3.730	0.010*	a a b b
Tem	1	0.179	0.684		0.533	0.486		1.300	0.287		11.293	0.134	
Tem*pH	1	0.254	0.628		0.488	0.505		0.758	0.409		0.268	0.618	
*hsp70*													
pH	1	3.765	0.088*	a a b b	10.662	0.910		1.570	0.246		1.887	0.207	
Tem	1	1.351	0.058*	a b a b	2.927	0.216		3.450	0.100		8.740	0.179	
Tem*pH	1	0.881	0.029*	a b b b	3.227	0.011*	a b b b	7.030	0.375		1.975	0.198	

Two-way repeated measures analysis of variance (ANOVA) significant at the *p*<0.1 level is indicated by asterisks. Results of multiple (Dunn-Sidak test) comparisons are given.

Means not sharing the same superscript are significantly different (p<0.1). (1) 8.10 T, (2) 8.10 T+3, (3) 7.70 T, (4) 7.70 T+3.

**Figure 2:**
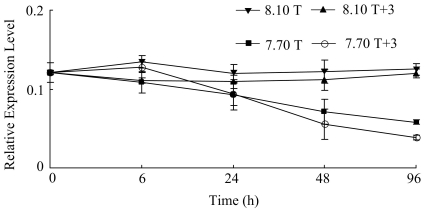
Real-time PCR analysis of expression of *calmodulin* in response to elevated temperature and declined pH.

The level of *calmodulin* did not change markedly during hours 0–96 in 8.10 T and 8.10 T+3 (*p*>0.1). In conditions 7.70 T+3 and 7.70 T, the level decreased significantly during hours 24–96 (*p*<0.1) (Table 2). The expression of *calmodulin* was affected significantly by pH and pH×temperature interaction at hours 48 and 96 (*p*<0.1) (Fig. 2). *Post hoc* analysis indicated that the expression of *calmodulin* showed significantly difference between condition 8.10 T, 8.10 T+3 and 7.70 T+3, 7.70 T (Table 3).

**Figure 3:**
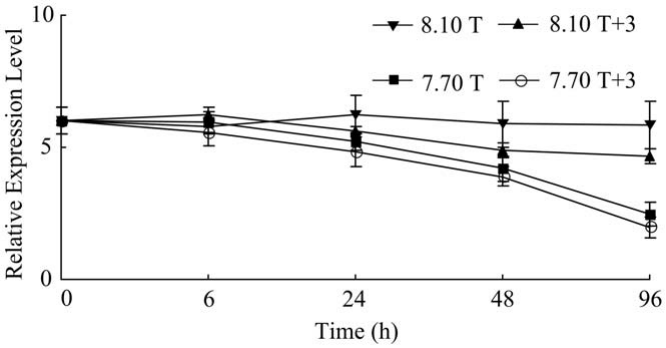
Real-time PCR analysis of expression of *nacrein* in response to elevated temperature and declined pH.

**Figure 4:**
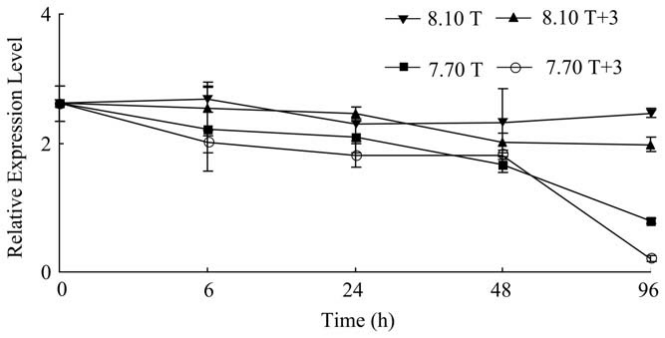
Real-time PCR analysis of expression of *she-7-F10* in response to elevated temperature and declined pH.

The expression level of *nacrein* at hours 24, 48 and 96 showed significantly difference from the level of time 0 in all the four treatments (*p*<0.1) (Table 2). ANOVA demonstrated a significant effect of pH and pH×temperature interaction on the expression of the *nacrein* gene on hour 96 (*p*<0.1) (Fig. 3). Significant difference was observed between condition 8.10 T, 8.10 T+3 and 7.70 T+3, 7.70 T (Table 3). The variations in the expression level of *she-7-F10* in the four treatments during the experiment were similar to those of *nacrein* (*p*<0.1) (Table 2). The expression level of *she-7-F10* was affected significantly by pH at hours 48 and 96 (*p*<0.1) (Fig. 4). The levels of expression were significantly different between conditions 8.10 T, 8.10 T+3 and conditions 7.70 T, 7.70 T+3 (Table 3).

**Figure 5:**
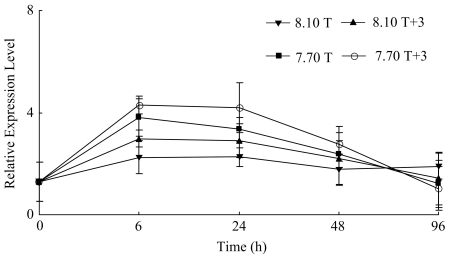
Real-time PCR analysis of expression of *hsp70* in response to elevated temperature and declined pH.

The expression level of *hsp70* increased significantly to a higher level on hours 6 and 24 in condition 8.10 T, 8.10 T+3, 7.70 T and 7.70 T+3, and then decreased markedly (*p*<0.1) (Table 2). The expression of *hsp70* was significantly affected by temperature, pH, temperature×pH interaction on hour 6, and by temperature×pH interaction on hour 24 (*p*<0.1) (Fig. 5, Table 3).

## Discussion

In the present study, environmental parameters that could affect the comparison between treatments were monitored. The gentle bubbling protocol maintained the seawater carbonate buffer system in a relatively stable condition in each treatment throughout the experiment. The seawater was saturated with respect to aragonite and calcite in each treatment. This demonstrates that the high seawater renewal rate used, successfully prevented any “aquarium” effect. We are, therefore, confident that *P. fucata* responses were only caused by pH and temperature treatment.

This study examined expression changes in genes of *aspein*, *calmodulin*, *nacrein* and *she-7-F10* in *P. fucata* associated with calcification under low pH and high temperature conditions. Shell and pearl formation of *P. fucata* needs a large amount of calcium and biomineralization implies the transport and control of calcium ions [35]. The present study showed that pH was the driving factor influencing levels of mRNA transcript for *aspein*, *calmodulin* and *nacrein*, whereas temperature aggravated the sensitivity of *P. fucata* to pH. The expression level of *she-7-F10* was affected significantly by pH only, suggesting that different genes respond differently to elevated temperature and declined pH. The down regulation of *aspein*, *calmodulin*, *nacrein* and *she-7-F10* was in accordance with Todgham and Hofmann [36], who found down regulation of calcification genes when sea urchin larvae of early prism stage were exposed to *p*CO_2_ values of 543 μatm. In contrast to these results, CO_2_ induced seawater acidification down to pH of 7.5 and 7.25 caused a compensatory increase in transcript levels of a range of calcification genes (msp130, SM30) in 3 day old *Paracentrotus lividus* larvae [37]. Zippay and Hofmann [38] showed that decreased pH did not affect the expression pattern of two shell formation genes at any of the abalone larval stages. The differences in results suggested that given no differences in methodology that responses may be due to adaptive capacity of a species to changing climatic conditions which differed between populations with a large geographic distribution.

The decrease in expression level of *aspein*, *calmodulin*, *nacrein* and *she-7-F10* may be linked to the decrease in calcification rate. The high sensitivity of these genes to declined pH was consistent with the general consensus on the negative relationship between *p*CO_2_ and calcification of marine bivalves [39]. If the elevated temperature and declined pH are maintained for longer, this decrease could trigger a cascading effect on shell growth and pearl formation of *P. fucata*. Considering the down regulation of calcification related genes observed in this study, it is possible that disturbances in the balance between the coordinated production of mineral and organic matrix could affect the composition and mechanical properties of the shell and pearl. This will further increase the ratio of low-quality pearls. When investigating the microstructure of larval spicules, Clark et al [40]. noted eroded surface structures in two out of four sea urchin species at CO_2_ acidified seawater of pH 7.70.

Living systems have evolved a variety of strategies to respond to external or internal environmental challenges. While these responses are often behavioral or metabolic, a powerful mechanism widely employed to maintain cellular homeostasis under stress is the adjustment of gene expression [36]. Bivalves will be affected by ocean warming and acidification predicted under climate change scenarios, and only those endowed with sufficient defense mechanisms will be able to survive. Expression of *hsp70* is frequently used as a component of physiological mechanisms through which bivalves cope with environmental challenges [41]. The present study showed that in the warming and acidified seawater conditions, activation of the heat shock response occurs in *P. fucata*. It was worth noting that combined exposure to elevated temperature and declined pH resulted in up regulation of *hsp70*, suggesting that elevated *p*CO_2_ aggravated the sensitivity of *P. fucata* to temperature. The interactive effects of temperature and *p*CO_2_ had already been reported in other studies. Anthony et al. reported higher reductions in algal calcification rate at high temperature (28–29°C; −190%) than at low temperature (25–26°C; −130%) under elevated *p*CO_2_ (1000–1400 ppm) relative to control conditions [42]. Reynaud et al. [15] reported that elevated *p*CO_2_ had no effects on coral calcification rates at 25°C, but caused a significant calcification decrease at 28°C. The up regulation of *hsp70* seemed to be consistent with the suggestion of O'Donnell et al. [43] that the gradual accumulation of *hsp70* might acted as a buffer against subsequent heat stress and support increased stress protection in gradually warming and acidified environments. The heat shock response was an energy consuming process. A shift to anaerobiosis as a result of thermally induced hypoxia in marine bivalves will caused metabolic depression and consequently a reduction in ATP turnover. If this was the case, there might be an effect on the expression of *hsp70*. Liu et al. [44] found that the clearance, respiration, and excretion rates of *P. fucata* decreased under low pH conditions. Thus, decrease in the expression of *hsp70* on hours 48 and 96 in the present study, may be related to decrease in the energy budget to meet the energy demand for *hsp70* synthesis. However, the response of *hsp70* to low pH and high temperature over long-term scales is still unknown and need further studies.

This study examined the short-term acute responses of *P. fucata* to seawater acidification. This leaded to some limitations for the results of the experiment. In the context of the results of this study, it is likely that this physiological stress was a result of the acute shock of transfer to treatment pH and temperature levels, and not evidence of physiological stress caused by long-term seawater acidification. These results provide fundamental information for the response of marine organism to CCS. However, further studies are needed to evaluate responses to elevated pCO_2_ and seawater temperature of marine organisms over longer time-scales.

In summary, our study showed that acidified and warming seawater resulted in a significant down regulation of calcification related genes, and the interaction between declined pH and elevated temperature caused up regulation of *hsp70* in *P. fucata*. The potential economic implications of this study for aquaculture industries include reduced growth rate of the pearl oyster and reduction in yield of the pearl. Our approach provides molecular elements on the response of *P. fucata* to CCS and projected climate change.

## Materials and Methods

### Seawater acidification and experimental design

One declined pH level (7.70), one ambient pH level (current level of 8.10), one elevated temperature (+3°C) and one ambient temperature (27°C) were selected for the study, based on projections by IPCC [2], [3] for the year 2100. Three replicates were set up for each temperature×pH treatment. Seawater was collected from Daya Bay Station, Chinese Academy of Sciences, on the southern coast of China (23°31′–24°50′N and 113°29′–114°49′E, average natural seawater pH 8.10±0.03, salinity 33±0.5‰). Water temperatures in elevated temperature treatment were maintained to 30±0.5°C using external chillers or unmanipulated for the ambient control. Temperatures were measured three times daily with a mercury thermometer. The pH in the acid seawater group (pH 7.70) was regulated by bubbling CO_2_ gas into the seawater until the desired pH was reached. The pH of each level in the experimental chamber was checked three times daily before and after water exchange to ensure stability throughout the experiment using a pH meter (PHS-3E Rex Instrument) calibrated with NBS standard buffers. With this high frequency of pH checks, we were able to sustain the targeted pH values with only a small range of variation over the course of the study (8.10±0.05 and 7.70±0.05). TA in each experimental tank was determined by potentiometric titration [45], [46] at hours 0, 48 and 96 before water exchange. The saturation states for aragonite, calcite and *p*CO_2_ values were determined from TA, pH_NBS_ and salinity data using CO2SYS [47] with the constants supplied by Mehrbach et al. [48] refitted by Dickson and Millero [49] and the KSO_4_ dissociation constant from Dickson [50].

### Animal collection and acclimation

Animals of similar sizes (shell height 48.92±1.41 mm) for the experiment were collected from the major pearl oyster growing area in Daya Bay Station (23°31′–24°50′N and 113°29′–114°49′E) (temperature 27±0.5°C, pH 8.10±0.05). This sampling procedure was done to ensure that the effects of ocean warming and acidification on gene expression patterns were reflective of the response of pearl oyster in their main area of distribution. The animals were selected and cleaned off epibionts, then acclimated in one 500 L aquarium at ambient seawater temperature (27±0.5°C) and pH (8.10±0.05) for one week prior to experimentation. This temperature was optimal for growth of *P. fucata* on the southern coast of China. They were fed daily with *Platymonas subcordiformis* at the satiation feed rate. Excess food and feces were removed by siphoning from the bottom of the aquarium, and fresh filtered seawater (salinity 33±0.5) was added into the aquarium every day.

After the acclimation period, animals were randomly assigned in twelve75-L aquariums (about 50 individual per aquarium) and maintained in four conditions: (1) ambient pH (8.10) and ambient temperature (27°C) (8.10 T, control condition), (2) ambient pH and elevated temperature (8.10 T+3), (3) declined pH (7.70) and ambient temperature (7.70 T), and (4) declined pH and elevated temperature (7.70 T+3). Each condition has three replicates. The header tanks were continuously bubbled with air to aid mixing and to maintain dissolved oxygen (DO)>90%. The experiment lasted for four days. The animals were fed daily with *Platymonas subcordiformis*. To ensure that similar conditions prevailed in each aquarium, except for the carbonate chemistry, water changes were performed daily in the aquariums. Seawater pH (7.70) of the low pH treatment was manipulated by bubbling CO_2_ gas into 100 L header tank. Water from the header tank was gravity-fed to replicate aquariums of pH 7.70. There was an individual header tank for each experimental aquarium. Water temperature was warmed to the required temperature (+3°C), or unmanipulated for the ambient control. To ensure no substantial change in pH and temperature occurred within experimental aquariums, pH and temperature in each aquarium were measured immediately after water changes. The pear oysters from all groups were sampled on hours 0, 6, 24, 48 and 96. On each sampling time point, 5 new individuals were randomly selected from each aquarium.

### Molecular analyses

Total RNA was extracted from the mantle tissue of *P. fucata* using the Mollusc RNA Kit (Omega Bio-Tek, Inc, Georgia, America) according to the manufacturer's instructions. The RNA quantity was analyzed on Thermo NanoDrop 2000 spectrophotometer (Thermo Fisher Scientific Inc, Wilmington, America) and quality was checked by gel electrophoresis. Then, high-quality total RNA was reverse transcribed to cDNA using the PrimeScript™ reagent kit (TaKaRa) following the manufacturer's instructions.

Quantitative real-time PCR reactions were carried out on Roche LightCycler480 thermal cycler (Roche Applied Science, Germany). The amplifications were performed in triplicate in a total volume of 10 µl containing 5 µl of 2× SYBR Premix Ex Taq™ (TaKaRa), 2 µl of diluted cDNA, 0.2 µl of each primer (10 µM) and 2.6 µl of double-distilled water. The cycle conditions were as follows: 1 cycle of 95°C for 10 s, 45 cycles of 95°C for 5 s, 60°C for 25 s, and 80°C for 1 s, 1 cycle of 95°C for 1 s, 65°C for 15 s, and 60°C for 1 s, 1 cycle of 40°C for 1 s. The specificity of the PCR amplification was verified from the melting curve. The housekeeping gene *gapdh* was selected as references for the calculation of relative expression levels of the genes. Primers sequences of *calmodulin*, *nacrein*, *aspein*, *she-7-F10*, *hsp70* and *gapdh* in *P. fucata* were outlined in Table 4.

**Table 4:** Primers sequences of genes used in real-time PCR analysis.

Gene	Acession no.	Primer sequence (5′-3′)
*she-7-F10*: F	EU177506	GAGGTCTCGGTGGAGTAAGTGG
*she-7-F10*: R		AACACTGCCGAGTCCTCCTAAT
*aspein*: F	AB094512	TTCATTTCGCTCTTTCAACCAG
*aspein*: R		GCATCCGAAGAACAAAGTTTTT
*calmodulin*: F	AY341376	TGACGGTGACGGACAGGTTA
*calmodulin*: R		GTTTGACAAAATTGCGTTTATGAC
*nacrein*: F	D83523	TGTTCATCTAACACCGGAGATG
*nacrein*: R		TGAAGAACCCTTCTTGACACCT
*hsp70*: F	EU822509	TTTGACTTGGGAGGAGGAACC
*hsp70*: R		CTCACCATTCTGTTGTCAAAGTCC
*gapdh*: F	AB205404	TCTGCTGATGCTCCTATGTTTG
*gapdh*: R		CGTTGATTATCTTGGCGAGTG

### Statistical analysis

To avoid pseudoreplication, all variables measured were averaged for 5 pearl oysters in each aquarium, and these aquarium means were used in all statistical analyses. All data were tested for homoscedasticity and normality and were log-transformed if necessary. The dependent variables (expression levels of the genes) at each time point were analyzed by two-way repeated measures ANOVA with temperature and pH as fixed factors. Dunn-Sidak tests followed ANOVA were used to assess differences among treatments and among different time point. Tank was analyzed as a factor by one-way ANOVA to assess tank effects. Significance was set at *p*<0.1 for all tests. The software SPSS 16.0 was used for analyses.
